# Extracellular vesicle-linked vitamin B_12_ acquisition via novel binding proteins in *Bacteroides thetaiotaomicron*

**DOI:** 10.1042/BCJ20253340

**Published:** 2025-12-05

**Authors:** Rokas Juodeikis, Robert Ulrich, Charlea Clarke, Michal Banasik, Evelyne Deery, Gerhard Saalbach, Bernhard Kräutler, Simon R. Carding, Michael A. Geeves, Richard W. Pickersgill, Martin J. Warren

**Affiliations:** 1 Quadram Institute Bioscience, Norwich, NR4 7UQ, United Kingdom of Great Britain and Northern Ireland; 2 School of Biosciences, University of Kent, Canterbury, CT2 7NZ, U.K.; 3 School of Biological and Behavioural Sciences, Queen Mary University of London, London, E1 4NS, United Kingdom of Great Britain and Northern Ireland; 4 John Innes Centre, Norwich, NR4 7UH, U.K.; 5 Institute for Organic Chemistry, University of Innsbruck, Innsbruck, A-6020, Austria; 6 University of East Anglia, Norwich, NR4 7TJ, U.K.

**Keywords:** bacterial extracellular vesicles, cobalamin, microbiome, B_12_-binding protein, nutrient acquisition

## Abstract

Vitamin B_12_ (cobalamin) and related cobamides are essential cofactors for many gut bacteria, yet their acquisition requires complex uptake systems due to limited availability. In the human gut commensal *Bacteroides thetaiotaomicron*, cobamide uptake is mediated by multiple operons encoding outer membrane proteins, transporters and uncharacterised lipoproteins, some of which are incorporated into bacterial extracellular vesicles (BEVs). Here, we advance the functional and structural understanding of this cobamide acquisition system by examining previously uncharacterised features. Bioinformatic and promoter–reporter analyses revealed four uptake operons, including novel genes we designate *btuK, btuJ, btuL and btuX*, with evidence for internal promoters and riboswitch regulation. Recombinant expression and binding assays identified ten cobamide-binding proteins, including three novel lipoproteins (BtuK1, BtuJ1 and BtuJ2). Biophysical measurements demonstrated affinities in the nano- to picomolar range, with BtuJ proteins displaying exceptionally tight binding. High-resolution crystal structures of BtuJ1 and BtuJ2 revealed an augmented β-jelly-roll fold, with conserved tyrosine residues forming a ‘halo’ around the corrin, suggesting a conserved binding mechanism within the IPR027828 protein family. Comparative proteomics of cells and BEVs under cobamide starvation showed selective enrichment of BtuJ and BtuL in BEVs. Functional assays demonstrated that BEV-mediated cobamide uptake depends specifically on BtuJ1 and BtuJ2, whereas BtuL promotes early-phase BEV release. These findings establish the BtuJ proteins as critical BEV-associated cobamide-binding components, provide structural insights into their tight binding, and suggest a model where BEVs act analogously to siderophores, capturing cobamides for delivery to cells. This work highlights the central role of BEVs in microbial nutrient competition.

## Introduction

**Figure 1:**
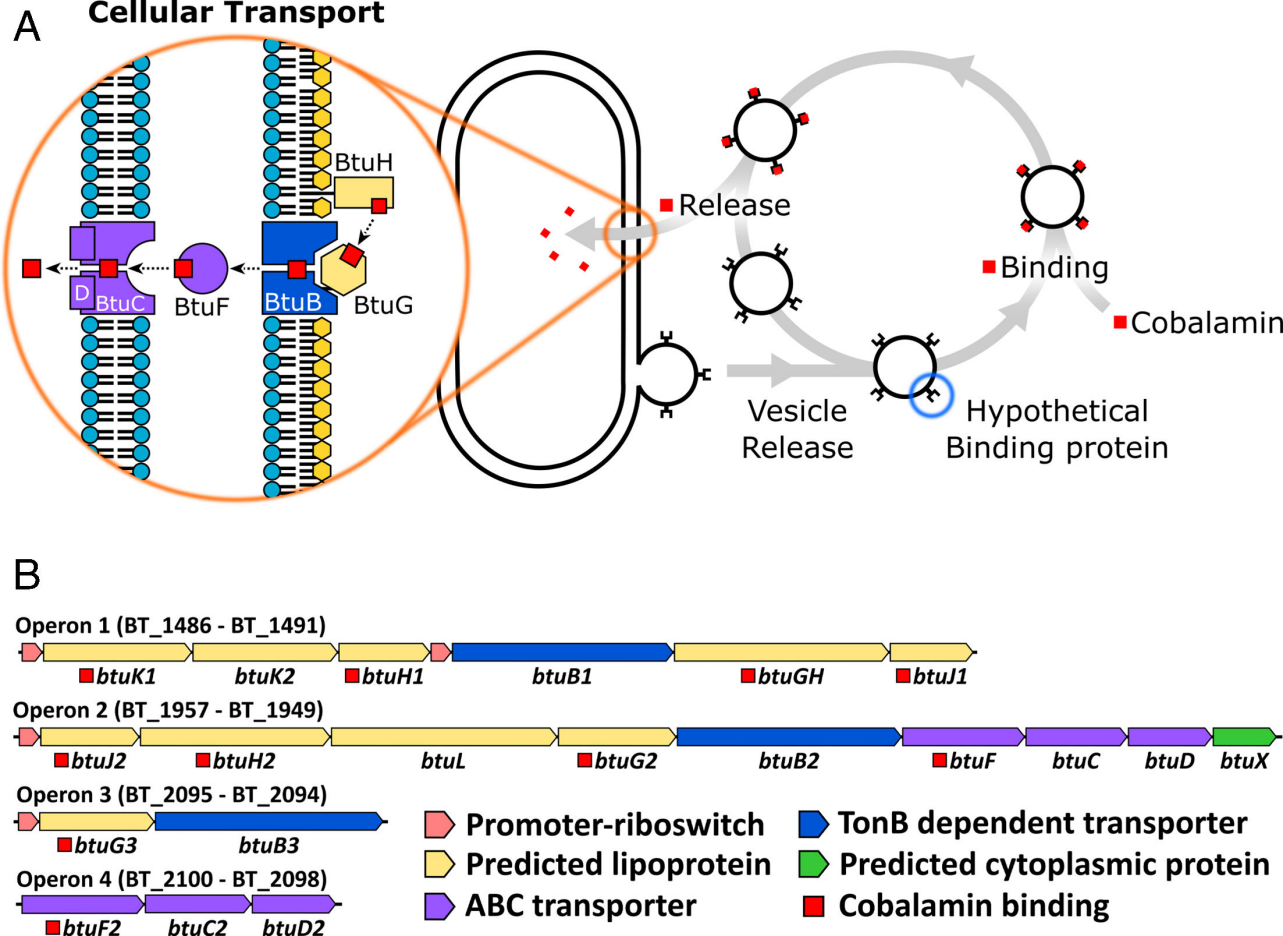
Vesicle role in nutrient acquisition and cobalamin uptake operons in *Bacteroides thetaiotaomicron* VPI-5482. **(A**) Previously proposed model of cobalamin uptake. Hypothetical proteins on extracellular vesicles (highlighted in blue) function analogously to siderophores, binding nutrients and delivering them to the cell in a receptor-dependent manner. Uptake across the outer membrane is mediated by the BtuBG complex, while transport across the inner membrane occurs via the BtuFCD complex, as described previously (highlighted in orange). (**B**) Panel shows the genetic elements involved in cobamide uptake. The operons are regulated by a cobalamin riboswitch, apart from operon 4. Notably, operon 1 contains an additional internal promoter site. Cobamide binding annotations are based on this publication.

Nutrient acquisition underpins bacterial metabolism, growth and adaptation to changing environments. In addition to conventional uptake mechanisms, bacteria produce extracellular vesicles (bacterial extracellular vesicles, BEVs) that not only transport enzymes and signalling molecules but also play a role in nutrient capture [1]. BEVs can sequester essential resources, such as iron and cobamides [2], from competing organisms and deliver them to the bacterial cell [1,3,4] (Figure 1A). Cobamides, corrinoid compounds containing a central cobalt ion within a corrin ring [2,5], are among the most critical of these nutrients. Acquisition of cobalamin (vitamin B_12_) is essential for *Bacteroides thetaiotaomicron*, a dominant commensal of the human gut [3,6–10]. Since this organism lacks the ability to synthesise cobalamin *de novo*, a highly complex and energetically demanding process [11,12], it relies on external sources of this vitamin to support essential metabolic functions, foremost of which is the activity of the B_12_-dependent methionine synthase (MetH) [3,13]. To compete effectively for cobamides in the gastrointestinal tract, *B. thetaiotaomicron* employs a sophisticated cobamide uptake system organised into multiple transport loci. These loci encode outer membrane cobamide-binding proteins and TonB-dependent transporters, as well as several poorly characterised lipoproteins (Figure 1B) [7]. The core components of the B_12_ transport and utilisation (Btu) system have been well characterised in molecular detail in Gram-negative bacteria [14,15]. In these organisms, BtuB functions as the TonB-dependent outer membrane transporter, while BtuC and BtuD form the ATP-dependent inner membrane importer, and BtuF serves as the periplasmic binding protein that mediates transfer between BtuB and the BtuCD complex (Figure 1A). In *B. thetaiotaomicron*, however, the *btu* operons are notably more complex, containing a greater number of genes whose functions are only beginning to be elucidated.

Of these associated gene products within these loci, recent work has identified high-affinity cobalamin-binding lipoproteins that are essential for nutrient capture and delivery to transporters [6,7,9,10]. The best characterised example, BtuG2, is required for the function of its co-expressed transporter BtuB, yet it is not found in other organisms known to use BtuB [6,7,9,10]. Another strong cobalamin binder, BtuH, is also encoded within these operons. Its biological role remains unclear, although structural studies suggest it can transfer cobalamin to BtuG [9]. This elaborate system underscores the competitive pressures shaping nutrient acquisition within the gut microbiota [7]. The presence of multiple cobamide-binding proteins suggests a co-operative, stepwise transfer mechanism that moves cobamides from the extracellular environment to the transporter, thereby enhancing efficiency and ensuring effective accumulation [6].

*B. thetaiotaomicron* has further adapted to this environment by producing BEVs, which expand its strategies for survival and competition. In our previous work, we demonstrated that its BEVs bind and specifically deliver cobalamin, effectively sequestering it from competing organisms, such as *Salmonella* [3,16]. Remarkably, these vesicles can also enter host cells, supplying essential micronutrients directly to the host [3]. As the gastrointestinal tract contains a chemically diverse pool of cobamides, including analogues in which the canonical lower ligand dimethylbenzimidazole is replaced by adenine or related derivatives, such versatility is advantageous. Distinct BtuB transporters allow *B. thetaiotaomicron* to acquire different cobamides with high selectivity, further strengthening its competitiveness in this nutrient-limited environment [7].

Despite these advances, the molecular details of cobamide uptake remain poorly understood. In particular, the precise roles of the various cobamide-binding lipoproteins encoded in the uptake operons, their interactions with structurally diverse cobamides and the mechanisms by which bound cofactors are released and transferred to transporters are unresolved. Equally unclear is how BEV-associated proteins contribute to cobamide acquisition and whether vesicle release integrates with the broader uptake system. Answering these questions is crucial for constructing a molecular framework of cobamide trafficking, from extracellular capture to intracellular delivery, and for understanding how this process confers a competitive edge in the gastrointestinal ecosystem.

In this study, we extend the characterisation of the cobamide uptake system in *B. thetaiotaomicron*. We identify additional, previously uncharacterised cobamide-binding lipoproteins within its uptake operons, define the proteins responsible for cobamide binding activity on BEVs, and establish a functional link between vesicle release and nutrient uptake. Together, our findings provide new structural and mechanistic insights into cobamide acquisition and reveal how BEVs integrate into bacterial nutrient acquisition strategies.

## Results

### Operon analysis

Previous work identified and characterised four operons located across three genetic loci in *B. thetaiotaomicron* VPI-5482 that are involved in the uptake of a range of cobamides (Figure 1B) [7]. Based on homology and gene number, we propose labelling the previously uncharacterised genes within these operons as *btuK1/2* (BT_1486/BT_1487), *btuJ1/2* (BT_1491/BT_1957), *btuL* (BT_1955) and *btuX* (BT_1949). Notably, except for *btuX*, all of these are predicted to encode lipoproteins, as indicated by the presence of a signal motif identified using SignalP 6.0 [17]. We further propose that BT_1486 has a misannotated start codon, suggesting methionine 36 is the correct start site. This is supported by the presence of a lipoprotein signal peptide starting at this sequence and the absence of the first 35 residues in the highly conserved BT_1487 gene. Details of our proposed operon annotations are available in .Supplementary Dataset 1.

Operons 1, 2 and 3 are predicted to be regulated by cobalamin riboswitches, with operon 1 containing an additional riboswitch localised upstream of *btuB1* (Figure 1B) [7]. These riboswitches are proposed to function by binding specific cobamides, inducing a structural rearrangement that leads to premature transcription termination and a consequent reduction in protein production [7,18]. In contrast, no riboswitch has been identified for operon 4.

**Figure 2:**
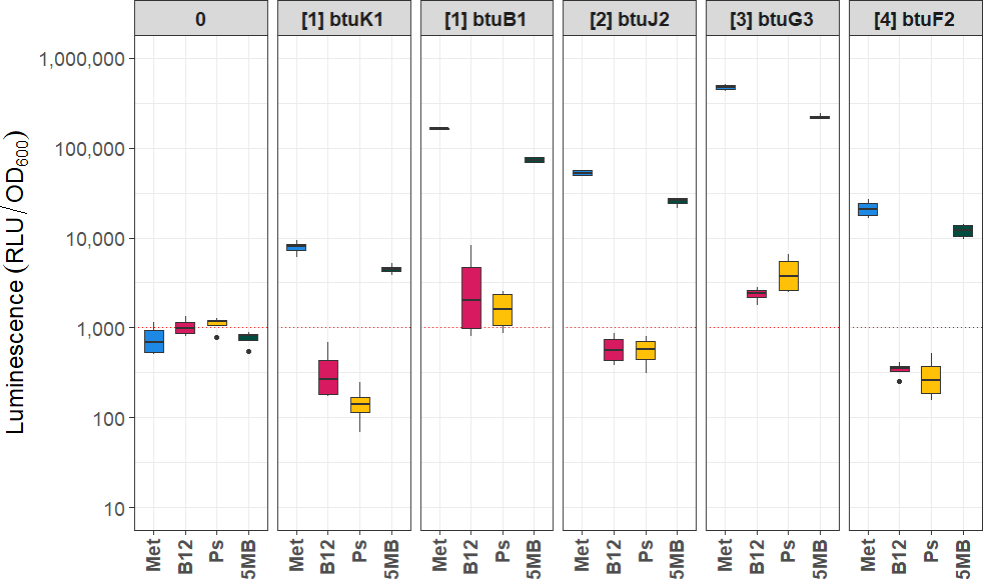
Regulation of identified promoters by different cobamides. Promoter activity was measured using a genomically integrated, single-copy luminescence reporter expressed from the indicated promoters. Promoter identities are shown above each plot, with 0 denoting the negative control. The associated operon is given in parentheses, and the numbering corresponds to the operons described in Figure 1B. The gene names listed on top of each panel indicate the first gene under control of the promoter. Each label therefore specifies the tested promoter along with its native ribosome-binding site. X-axis labels denote the compound added to the growth media: Met (400 µM L-methionine, positive control), B12 (cyanocobalamin), Ps (pseudocobalamin), and 5 MB (5-methylbenzimidazole cobamide). Across all promoters, B12 and Ps nearly abolished reporter activity, while 5 MB caused ~ 50% reduction. Boxplots represent four biological replicates; the red dotted line indicates the experimental noise threshold.

To confirm the regulation of these operons, we generated reporter constructs using a single-copy integration vector targeting the promoter regions, including the site upstream of *btuB1*. Each construct incorporated the downstream ribosome binding site, which was used to name the constructs, to drive translation. Nanoluciferase was used as the reporter gene [19]. The resulting reporter strains were analysed under conditions with three different cobamides, using methionine (Met) as a control (Figure 2). As expected, in the absence of cobamides and with methionine as the growth substrate, gene expression was induced. Among the operons, operon 3 exhibited the highest expression level, while the promoter upstream of operon 1 showed the lowest. Interestingly, the promoter upstream of *btuB1* within operon 1 demonstrated high levels of translation, confirming its role as an active promoter site. In the presence of cobalamin (B_12_) and pseudocobalamin (Ps), all tested promoters exhibited near-complete repression of gene expression. However, exposure to 5-methylbenzimidazole cobamide (5 MB) resulted in partial down-regulation, reducing protein production to approximately 50%.

### Cobalamin-binding lipoproteins

To gain deeper insight into cobamide uptake in *B. thetaiotaomicron* and explore the role of the recently identified novel B_12_-binding proteins encoded within the four operons in salvaging cobamides from the growth media, we first sought to identify all the cobalamin-binding proteins encoded within these regions, many of which are predicted to be lipoproteins. To achieve this aim, we recombinantly expressed each identified lipoprotein in *Escherichia coli*, replacing their N-terminal lipoprotein motif with a His-tag to facilitate purification. Proteins capable of binding cobalamin were identified based on their ability to retain the cofactor when bound to a nickel affinity column.

This approach identified a remarkable total of ten cobalamin-binding proteins encoded within the four operons (Figure 1B, red squares annotations). These include the previously identified BtuGH (formerly BtuG1), BtuG2/3 and BtuH1/2. Sequence analysis of BtuGH suggests that it represents a fusion of BtuG and BtuH domains. Upon dissecting this protein into its two component domains, we confirmed that both independently bind cobalamin. We also verified that the two predicted cobalamin ABC transporter periplasmic components (BtuF1 and BtuF2) are indeed cobalamin-binding proteins. Interestingly, BtuF2 contains a predicted lipoprotein sequence, though its functional significance was not explored in this study. Additionally, we identified three novel cobalamin binding proteins. Both BtuJ1 and BtuJ2 demonstrated B_12_ cobalamin binding, whereas BtuK1, but not BtuK2, was also able to bind cobalamin. This was unexpected, given the sequence similarity between BtuK1 and BtuK2. Finally, no cobalamin binding was observed for BtuL.

**Figure 3:**
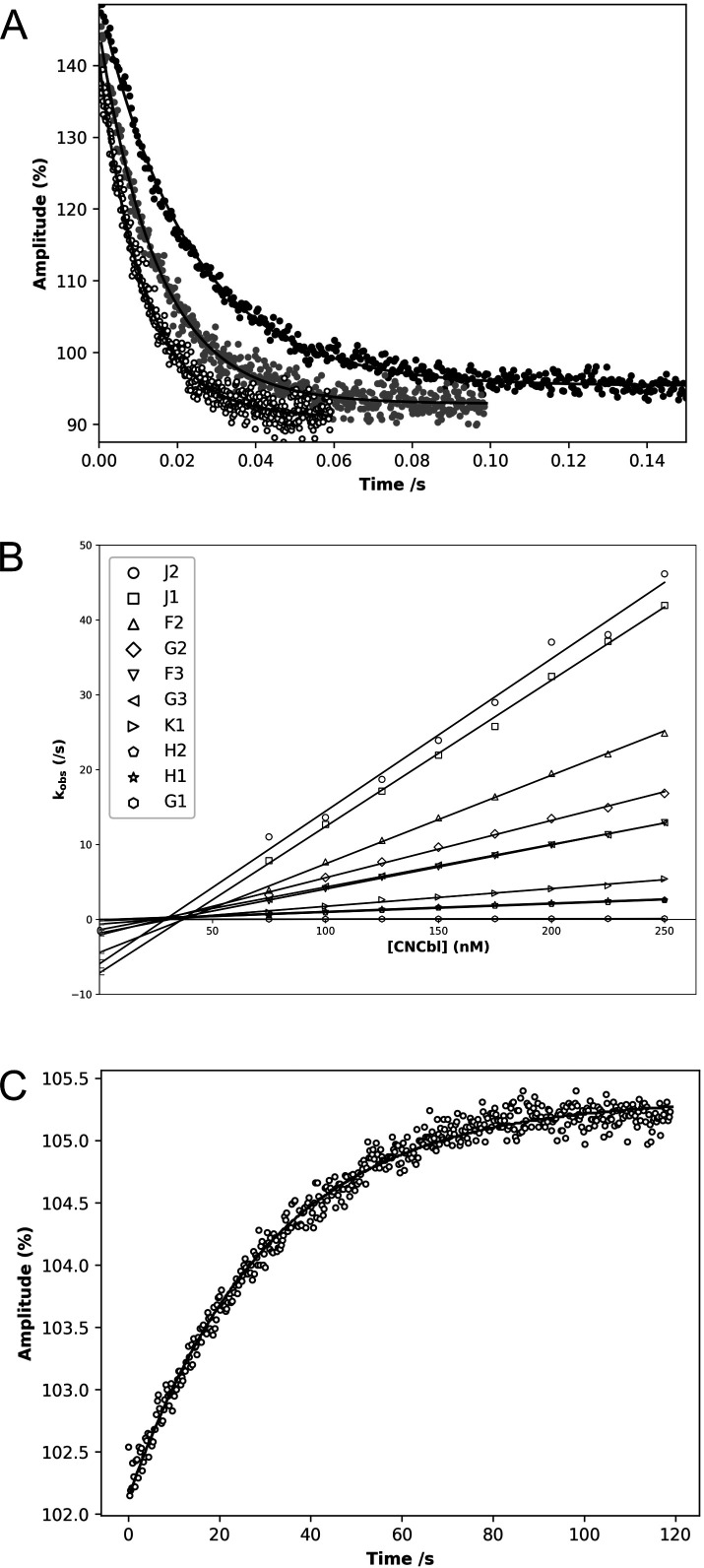
The binding kinetics of BtuJ1 and other B_12_ binding proteins. (**A**) Representative dataset to establish *K*on by stopped flow. The change in tryptophan fluorescence upon BtuJ1 binding to CN-Cbl using 25 nM BtuJ1. Black, gray and white circles are 75 nM, 150 nM and 225 nM CN-Cbl, respectively; from which *K*on can be determined to be 2.10 × 10^8^ default 0.07 M^-1^s^-1^. (**B**) Linear regression of Kobs for BtuJ1 and nine other proteins. *K*obs plotted against a series of CN-Cbl concentrations with 25 nM protein. A remarkable range of on rates is observed with BtuJ2 showing the fastest rate, where *K*on = 2.2 x 10^8^ M^-1^s^-1^, and BtuGH had the slowest rate of *K*on = 3.45 x 10^5^ M^-1^s^-1^. The key is ordered by decreasing Kon value. (**C**) *K*off determination. An example of the change in tryptophan fluorescence upon BtuJ2 displacing BtuJ1 from its complex with CN-Cbl, allowing a *K*off value of 0.032 s^-1^ to be determined.

To understand better the roles of these cobalamin-binding proteins in sequestering exogenous cobamides and facilitating their transport to the cellular cytoplasm, we investigated their binding kinetics, given previous reports of femtomolar binding affinities [10]. To achieve this, we employed stopped-flow spectroscopy and surface plasmon resonance (SPR) measurements to characterise their binding properties. Stopped flow measurements revealed that cobalamin binding to the Btu proteins led to a decrease in intrinsic tryptophan fluorescence, in some cases by as much as 50% (Figure 3A). Plots of the observed rate constants (*K*obs) as a function of cobalamin concentration were linear, allowing determination of the second-order association rate constant (*K*on) from the slopes (Figure 3B). The poorly defined intercepts indicated that *K*off values were much smaller than the slowest measured *K*obs, consistent with dissociation constants (*K*d = *K*off/*K*on) in the nanomolar range. In selected cases, *K*off could be determined directly by displacement assays, in which cyanocobalamin was released from Btu-cyanocobalamin complexes upon addition of excess competitor proteins with distinct fluorescence signatures. For example, displacement of cyanocobalamin from BtuJ1 by BtuJ2 yielded a *K*off of 0.032 s^-1^ (Figure 3C), corresponding to a *K*d of 0.16 nM. Among the Btu proteins, BtuJ1 and BtuJ2 displayed the fastest association rate constants (BtuJ1 *K*on = 2.10 x 10^8^ M^-1^s^-1^; BtuJ2 *K*on = 2.20 x 10^8^ M^-1^s^-1^), consistent with diffusion-controlled ligand binding (typically 1–10 × 10^8^ M^-1^s^-1^) [20,21]. Other *K*on values were generally one or more orders of magnitude lower, with BtuGH being markedly slower still (*K*on of 3.4 × 10^5^ M^-1^s^-1^). While stopped-flow analysis provided accurate estimates of rapid association rates, most dissociation rates were either poorly defined or not measurable under the experimental conditions.

SPR was employed to resolve the challenges in defining binding kinetics for these proteins, which exhibited both extremely rapid on rates and relatively slow off rates in the stopped-flow experiments. Measurements were performed on a Biacore T200 system, which provides high sensitivity, optimised fluidics and advanced data analysis capabilities (Supplementary Figure S1). In comparison with stopped-flow, SPR yielded slower apparent association rates, likely reflecting the effects of protein immobilisation on the sensor surface that can restrict binding dynamics (Table 1). Importantly, however, SPR allowed accurate determination of dissociation rates, which were consistently slower than those inferred from stopped flow. From these measurements, cyanocobalamin affinities were estimated to range from 0.037 nM for BtuGH to 11 nM for BtuH1. Given the particular strength of SPR for measuring dissociation events, these *K*off values are likely to provide more reliable estimates than those obtained by stopped-flow. By integrating the rapid *K*on values from stopped-flow with the more precise *K*off values from SPR, we infer that proteins such as BtuG2, BtuG3 and BtuK1 may bind cyanocobalamin with picomolar affinities. Accurately quantifying ultra-tight binding events remains notoriously difficult in biochemistry, since both rapid-mixing approaches and SPR are limited by temporal resolution, mass transport effects and the inability to reliably measure very slow dissociation rates. Hence, the data we present here demonstrate that these proteins bind with high affinity, but the interpretation is constrained by the inherent limitations of these methods.

**Table 1:** Summary of kinetics parameters obtained from stopped flow and SPR

	Stopped flow			Surface Plasmon Resonance		
Protein	*K*on (s^-1^M^-1^)	*K*off (s^-1^)	*K*d (M)	*K*on (s^-1^M^-1^)	*K*off (s^-1^)	*K*d (M)
BtuF	8.8 × 10^7^ ± 0.2	3.3 × 10^-1^ ± 9.8	3.8 × 10^-9^ ±11.1	ND	ND	ND
BtuF2	6.20 × 10^7^ ± 0.05	ND	ND	6.60 × 10^5^ ± 0.07	5.7 × 10^-3^ ± 0.04	8.6 × 10^-9^ ± 0.1
BtuGH	3.4 × 10^5^ ± 0.2	4.8 × 10^-3^ ± 4.2	1.4 × 10^-8^ ± 1.2	6.40 × 10^5^ ± 0.03	2.3 × 10^-5^ ± 0.2	3.70 × 10^-11^ ± 0.01
BtuG2	6.2 × 10^7^ ± 0.1	9.3 × 10^-1^ ± 16	1.5 × 10^-8^ ± 2.6	5.20 × 10^5^ ± 0.02	2.2 × 10^-5^ ± 0.1	4.2 × 10^-11^ ± 0.3
BtuG3	5.80 × 10^7^ ± 0.08	ND	ND	2.00 × 10^5^ ± 0.01	4.0 × 10^-5^ ± 0.1	2.00 × 10^-10^ ± 0.05
BtuH1	9.5 × 10^6^ ± 0.2	9.0 × 10^-2^ ± 10	9.5 × 10^-9^ ± 10.5	5.10 × 10^5^ ± 0.05	5.60 × 10^-3^ ± 0.03	1.1. x 10^-8^ ± 0.01
BtuH2	8.9 × 10^6^ ± 0.2	2.0 × 10^-1^ ± 7.2	2.3 × 10^-8^ ± 8.1	ND	ND	ND
BtuJ1	2.10 × 10^8^ ± 0.07	3.2 × 10^-2^ ± 0.2	1.5 × 10^-10^ ± 0.1	1.3 × 10^7^ ± 0.01	1.10 × 10^-2^ ± 0.01	8.46 × 10^-10^ ± 0.10
BtuJ2	2.20 × 10^8^ ± 0.03	ND	ND	ND	ND	ND
BtuK1	1.80 × 10^7^ ± 0.02	2.9 × 10^-1^ ± 5.0	1.6 × 10^-8^ ± 2.8	1.80 × 10^5^ ± 0.01	1.60 × 10^-5^ ± 0.08	8.9 × 10^-11^ ± 0.5

ND: not determined.

### The structures of BtuJ1 and BtuJ2 with bound cyanocobalamin

The previously uncharacterised BtuJ1 and BtuJ2 proteins share 23% amino acid sequence identity and belong to the IPR027828 protein family [22]. Recombinant BtuJ1, expressed without its first 26-residue lipidation signal sequence, was crystallised in complex with cyanocobalamin. The structure was solved using molecular replacement with the AlphaFold Molecular Replacement workflow in the CCP4i program suite (see Materials and Methods for references and details). The crystals belong to space group C2_1_ and contain two BtuJ1/cyanocobalamin complexes in the asymmetric unit. The structure was refined to 1.6 Å resolution with good stereochemistry, achieving R_work_ and R_free_ values of 0.191 and 0.216, respectively (data collection and refinement statistics are summarised in Supplementary Table S1). The two complexes in the asymmetric unit are structurally similar, with an RMSD of 0.095 Å across 199 equivalenced Cα atoms.

The structure of BtuJ2 was determined using the same workflow as for BtuJ1. BtuJ2 crystals belong to space group P2_1_2_1_2 and contain two molecules in the asymmetric unit. The structure was solved to 2.7 Å resolution, with reasonable crystallographic residuals and stereochemistry (Supplementary Table S1). The two copies in the asymmetric unit of BtuJ2 exhibit an RMSD of 0.127 Å across 211 equivalenced Cα atoms.

**Figure 4:**
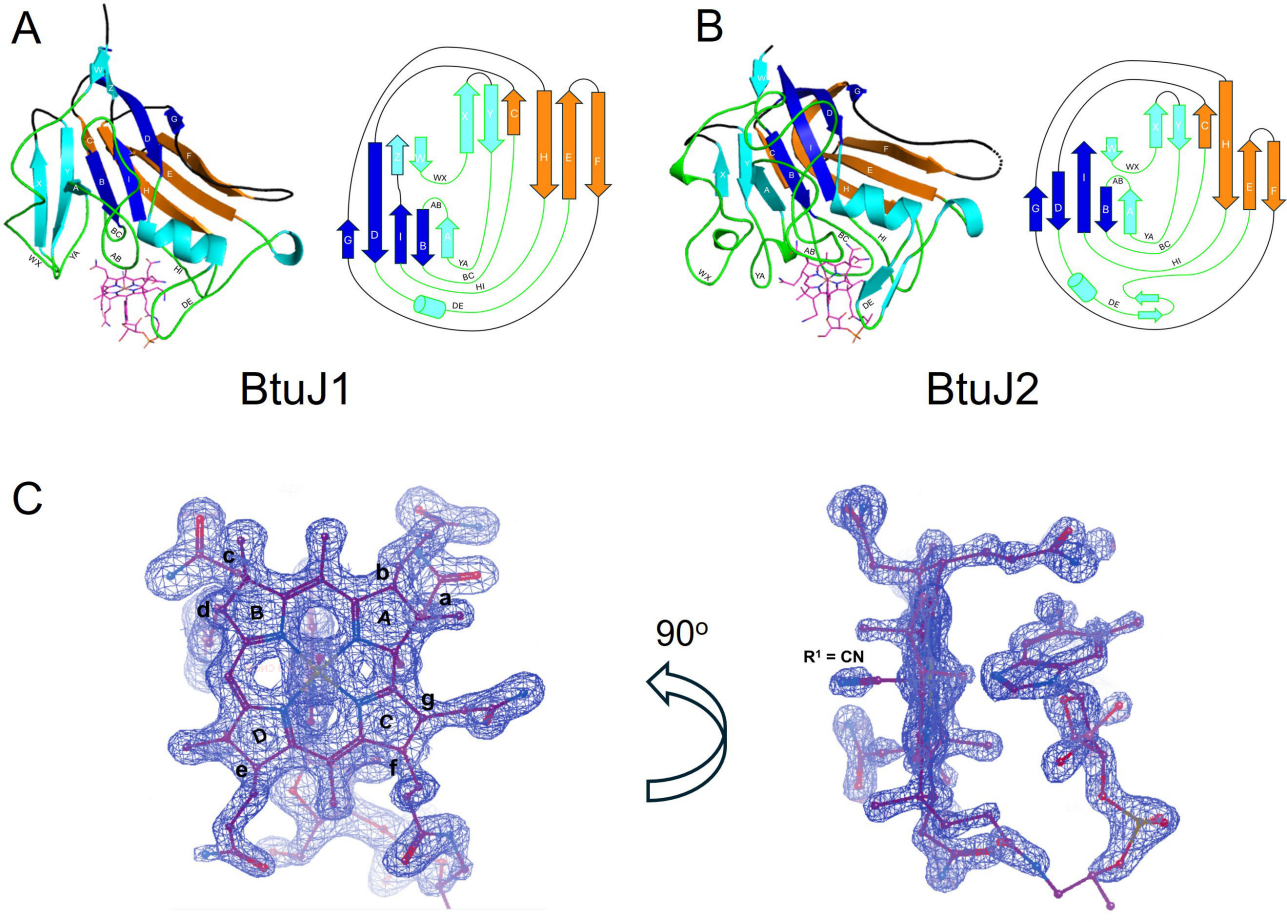
Crystal structure of BtuJ1 and BtuJ2 with bound cobalamin. The structure of BtuJ1 and BtuJ2 with cobalamin bound (PDB: 9FCT and 9I2L). **A.** The augmented β-jellyroll architecture of BtuJ1 with additional β-strands (and helix) in light blue. Four loops (AB, BC, DE, HI) are directly involved in binding to cobalamin. **B.** The architecture of BtuJ2 and its topology. There is considerable variation in β-strand length and loop structures compared with BtuJ1. **C.** Refined electron density for cobalamin bound to chain A of BtuJ1 in the crystal structure. Two orthogonal views of the electron density and cobalamin are shown. The binding site is shown in more detail in Figure 5.

BtuJ1 and BtuJ2 share a core jellyroll β-barrel structure, where the polypeptide chain forms eight β-strands organised into two four-stranded antiparallel β-sheets [23]. This core architecture is common in viral capsid proteins [24] and also proteins of cellular origin [25]. In BtuJ1 and BtuJ2, the core structure is augmented by three additional antiparallel β-strands at the N-terminal end of the jellyroll and an α-helix located between strands D and E in the traditional jellyroll nomenclature (BIDG and CHEF, Figure 4A and B). When comparing BtuJ1 and BtuJ2, they exhibit a common core architecture (RMSD of 4.0 Å across 110 equivalent Cα atoms) but display significant differences in their loop regions. The HI loop, which contains a conserved tyrosine residue at its terminus near the start of strand I, is the most structurally similar in these two proteins. This conserved loop is likely the key region involved in cobalamin binding.

Cyanocobalamin is well-resolved in both crystal structures, with evidence of binding shown for BtuJ1 in Figure 4C. The cobalamin-binding pocket is formed by four loops from the jellyroll structure, loops: AB, BC, HI and DE and two additional loops associated with the ancillary β-strands; AB and ZA (Figure 4A and B). Within the pocket, the adenosyl group of the lower ligand faces outward, extending into the solution. Both binding pockets have sufficient space to accommodate the bulkier adenosyl group of Ado-Cbl, and binding would displace water molecules, suggesting an entropic contribution to tight binding of Ado-Cbl.

**Figure 5:**
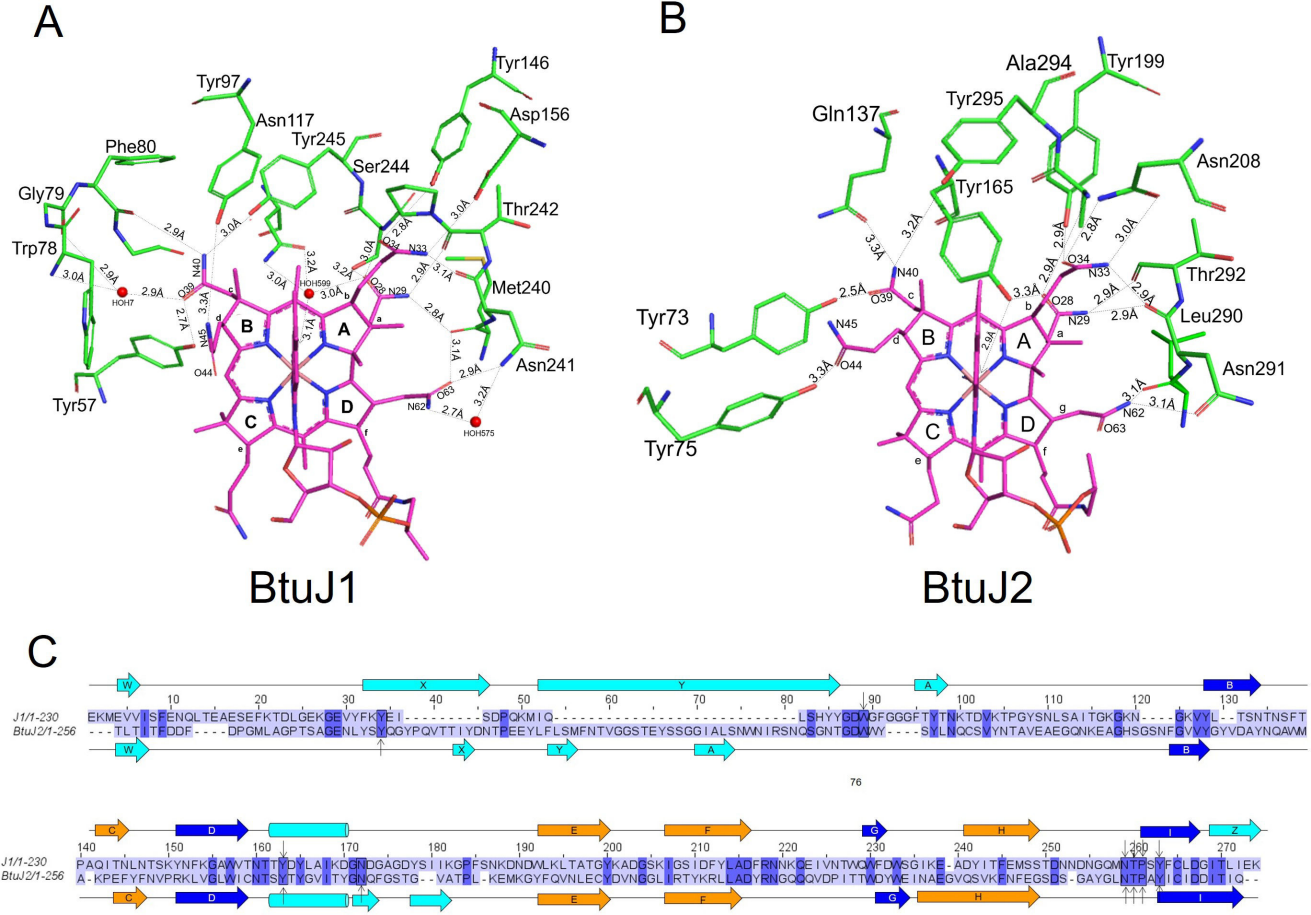
Hydrogen bonds to cobalamin and sequence conservation between BtuJ1 and BtuJ2. **A**. Cartoon showing the residues of BtuJ1 that form hydrogen bonds to the cobalamin sidechains (*a*, *b*, *c* and *d*). **B**. As panel A, but for BtuJ2. There is an approximately equal mix of mainchain and sidechain hydrogen bonds to cobalamin in both proteins. **C**. Sequence alignment of ButJ1 and BtuJ2 which have 23% sequence identity (conserved residues in blue block). Residues in the black boxes are conserved residues hydrogen bonding to cobalamin in the crystal structures of BtuJ1 and/or BtuJ2. The arrows indicate the protein making the hydrogen bond. The conserved hydrogen bonding residues are: Tyr146 in BtuJ1 and Tyr199 in BtuJ2, Asn291 and Asn241 (ButJ1/BtuJ2), Thr242 and Thr292 (BtuJ1/BtuJ2), Tyr245 and Tyr295 (BtuJ1/BtuJ2). Coloured arrows represent β-strands and rectangles α-helices, and labelling corresponds to the topology diagrams shown in Figure 4.

The binding is predominantly characterised by shape complementarity between the pocket and the corrin ring, complemented by hydrogen-bonding. These interactions involve sidechain polar groups as well as mainchain carbonyls. When the cobalamin molecules are superimposed, the core architecture of BtuJ1 and BtuJ2 aligns well, highlighting similarities in the binding residues (Figure 5).

A notable feature in both proteins is a halo of tyrosine residues that contribute hydrophobic interactions as well as hydrogen bonding to the corrin ring’s side chains. Conserved tyrosine residues in analogous positions include (BtuJ2 residue number in parentheses): Tyr 97 (134) on AB loop, Tyr 146 (199) at N-terminus of the helix within the DE loop (hydrogen bonding the *b* sidechain of the corrin ring), and Tyr 245 (295) at the end of the HI loop/start of strand I (hydrogen bonding to the *c* sidechain of the corrin ring).

An interesting distinction is observed in BtuJ1, where Trp 78 is involved in interactions with the dimethylbenzimidazole group of cobalamin. In BtuJ2, this tryptophan is replaced by a pair of tyrosine residues, resulting in a modification of the interaction with the cobalamin side chain (*d*), which was sandwiched between the dimethylbenzimidazole group and Trp 78 in BtuJ1. Overall, while the shape, hydrophobicity and hydrogen bonding characteristics of the binding site are retained in both structures, the specific residues contributing to these interactions differ, reflecting subtle variations in binding mechanisms between BtuJ1 and BtuJ2.

### IPR027828 protein family analysis

The major binding motif (MNTPSY in *B. thetaiotaomicron*) includes the highly conserved Tyr245, a hallmark across the IPR027828 protein family [22]; also known as Domain of Unknown Function, DUF4465 [26]. Proline and threonine residues occur frequently within this motif, and the tyrosine is universally present at the end of loop 6 (L6). This loop is a distinct and recognisable feature in all sequences for which AlphaFold models are available. Many of the interactions with the cobalamin ring are mediated by mainchain carbonyl oxygens, suggesting that this cobalamin-binding site is likely conserved across the IPR027828 protein domain family. To understand further the localisation of IPR027828 family members, we analysed their targeting signals using SignalP 6.0 [17] and examined co-occurrences with InterPro domains associated with protein targeting. Out of 824 non-redundant sequences investigated, 38% contained InterPro annotation for a C-terminal extracellular targeting motif (229/824 IPR026444, 88/824 IPR013424), 33% contained a lipoprotein targeting sequence (275/824) while 21% contained a periplasmic targeting sequence (170/824). Notably, none of these targeting motifs co-occurred within individual sequences. Based on these observations, we propose that the IPR027828 family primarily consists of extracellular cobalamin-binding proteins, reflecting their likely role in nutrient acquisition and transport.

### Comparative cell and BEV proteomics under cobamide starvation

In our previous work, we demonstrated that *B. thetaiotaomicron* BEVs can bind and deliver a range of cobamides [3]. To determine which of the identified cobalamin-binding proteins are involved in this BEV-mediated process, we conducted comparative proteomic analyses of cells and BEVs produced in cobamide-containing and cobamide-free media (Table 2 **; Supplementary Datasets S2 and S3**). As expected, due to the regulation of these operons by cobalamin riboswitches, most of these proteins were significantly up-regulated in both cells and BEVs when cobalamin was absent from the media. Similar regulatory patterns have been reported in recent studies [6]. Notably, the protein up-regulation profiles differed between cells and BEVs. The highest relative enrichment in BEVs was observed for BtuK2, BtuH1, BtuJ1 and BtuL, suggesting selective incorporation of these proteins into BEVs. Interestingly, BtuJ2 was undetectable in cells but showed significant up-regulation in BEVs, highlighting its potential specialised role in vesicle-mediated cobamide transport.

**Table 2:** Proportional upregulation of cobamide uptake operon proteins in cells and BEVs produced in B12 free media. ND indicates no signal detected. Statistical significance: *** p < 0.001, ** p < 0.01, * p < 0.05, ns (not significant) p > 0.05. Signal sequence from SignalP: Lipidation - secretion & lipidation signal, Secretion - secretion signal, or none detected.

Protein	Gene	Signal sequence	Cell (Met/B12)	BEV (Met/B12)
*Encoded by operon 1*				
BtuK1	BT_1486	Lipidation	81.1 (***)	291.9 (***)
BtuK2	BT_1487	Lipidation	104.5 (***)	1000 (***)
BtuH1	BT_1488	Lipidation	123.5 (***)	1000 (***)
BtuB1	BT_1489	Secretion	12.2 (***)	79.7 (***)
BtuGH	BT_1490	Lipidation	12.5 (***)	21.8 (*)
BtuJ1	BT_1491	Lipidation	31.6 (***)	471.3 (***)
*Encoded by operon 2*				
BtuJ2	BT_1957	Lipidation	ND	27.3 (***)
BtuH2	BT_1956	Lipidation	102.4 (***)	259.9 (***)
BtuL	BT_1955	Lipidation	99.6 (***)	1000 (***)
BtuG2	BT_1954	Lipidation	74 (***)	158.8 (***)
BtuB2	BT_1953	Secretion	22 (***)	45.4 (**)
BtuF	BT_1952	Lipidation	208.8 (***)	112.2 (***)
BtuC	BT_1951	None detected	ND	ND
BtuD	BT_1950	None detected	13.9 (***)	8.1 (ns)
BtuX	BT_1949	None detected	5.8 (***)	ND
*Encoded by operon 3*				
BtuB3	BT_2095	None detected	4.8 (**)	47.3 (**)
BtuG3	BT_2094	Lipidation	47.9 (***)	118.1 (***)
*Encoded by operon 4*				
BtuF2	BT_2100	Lipidation	0.7 (ns)	0.5 (ns)
BtuC2	BT_2099	None detected	ND	ND
BtuD2	BT_2098	None detected	0.9 (ns)	ND

In our recent study, we characterised the dynamics of BEV production by *B. thetaiotaomicron*, demonstrating that BEVs released early during growth are enriched with specifically targeted lipoproteins, whereas those released later contain whole cellular components, likely as a result of cell lysis [16]. Using principal component analysis of the temporal proteomic data, we identified BtuK1, BtuK2, BtuJ1 and BtuL as major contributors to the BEV protein composition at distinct time points (Supplementary Table S2). This finding suggests that these proteins are selectively incorporated into specialised, non-lytic BEVs during the early growth phase.

### BtuJ delivers cobalamin via BEVs

**Figure 6:**
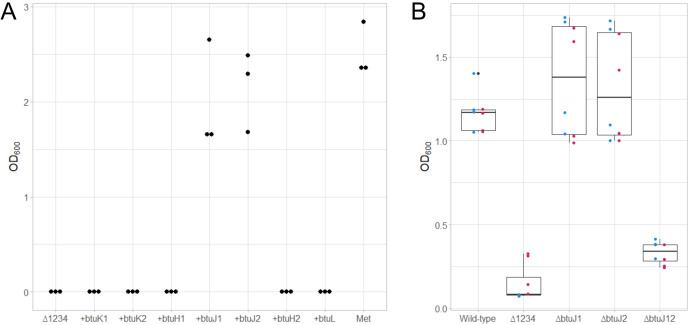
BtuJ1 and BtuJ2 are responsible for cobalamin delivery from *B. thetaiotaomicron* bacterial extracellular vesicles. BEVs from a range of generated *B. thetaiotaomicron* strains were used as the only source of cobalamin for wildtype *B. thetaiotaomicron*. **A**. Bioassay showing that either BtuJ1 or BtuJ2 is sufficient for cobalamin binding and delivery activity of BEVs. Δ1234 - *B. thetaiotaomicron* strain lacking all cobamide uptake genes; [ + gene] strains. Individual genes were reintroduced into Δ1234 genetic background; Met is the positive control containing 400 µM L-methionine. The bioassay was in triplicate with one biological BEV replicate. Results for additional genes available as Supplementary Figure S3. **B**. Growth recovery bioassay using cobalamin bound BEVs showing that either *btuJ1* or *btuJ2* are required for cobalamin binding and delivery activity. Wildtype used as the positive control; Δ1234 used as the negative control; *ΔbtuJ1, ΔbtuJ2, ΔbtuJ12* are specific *btuJ1, btuJ2* or double *btuJ1* and *btu2* knockout strains, respectively. The bioassay had four biological replicates of two biological replicates of BEV preparations indicated by coloured dots with boxplots showed in black.

To determine whether any of these proteins contribute to cobalamin-binding activity in BEVs, we generated strains specifically expressing *btuK1, btuK2, btuJ1, btuJ2, btuH1, btuH2* or *btuL* in a mutant background lacking all cobamide uptake operons. BEVs purified from these strains were saturated with cobalamin, thoroughly washed and provided to wildtype *B. thetaiotaomicron* cells as the sole source of cobalamin. The concentration of BEVs used was equivalent to that present in the late phase culture. Notably, only BEVs containing BtuJ1 or BtuJ2 served as an effective source for cobalamin (Figure 6A). We also tested BEVs derived from null mutant strains expressing *btuG* or *btuB* genes, which showed no significant binding activity (Supplementary Figure S3). These findings suggest that BtuJ1 and BtuJ2 are critical for cobalamin uptake via BEVs.

To validate this observation, we constructed *∆btuJ1*, *∆btuJ2* and *∆btuJ1/2* knockout strains of *B. thetaiotaomicron*. As before, BEVs from these strains were saturated with cobalamin, washed and used at biologically relevant levels to rescue growth in cobamide-free media (Figure 6B). BEV concentrations were equivalent to those found in late-phase cultures. While BEVs from individual knockout strains (∆*btuJ1* or ∆*btuJ2*) showed no significant effect on cobamide uptake, BEVs from the double knockout strain (∆*btuJ1*/*J2*) exhibited a nearly complete loss of cobalamin-binding activity. These results strongly support the hypothesis that BtuJ1 and BtuJ2 are the primary proteins responsible for cobamide binding in BEVs and their subsequent release to cells.

### Cellular elements required for BEV-cobalamin uptake

Significant progress has been made in understanding the minimal cellular requirements for cobalamin uptake; however, certain specific combinations of gene knockouts have not yet been explored [7]. Additionally, the role of BEV-associated cobalamin in this process remains unaddressed. To extend these previous findings and determine the cellular components necessary for utilising BEV-bound cobalamin, we generated a series of novel knockout strains in the wildtype *B. thetaiotaomicron* background. These included strains with targeted operon deletions, such as ∆12, ∆23, ∆134 and ∆1234, as well as a complex knockout strain (∆13 .J2.H2.L) lacking operons 1 and 3 along with *btuJ2*, *btuH2* and *btuL,* while retaining the promoter region of operon 2.

**Figure 7:**
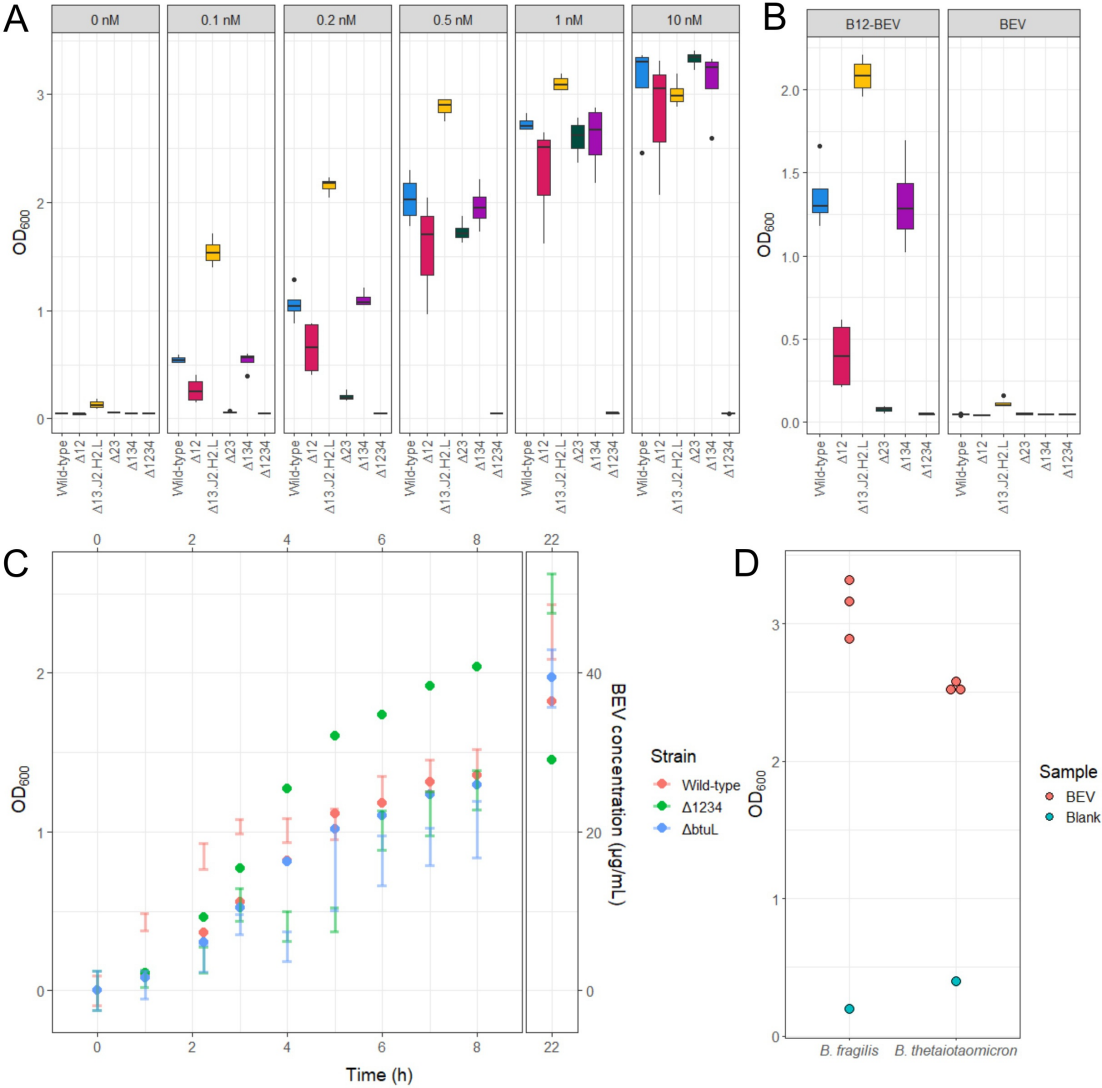
*Bacteroides* cobalamin uptake operon mutant analysis. **A.** Different *B. thetaiotaomicron* strain growth in the presence of varying cobalamin concentrations. Boxplots show four biological replicates. **B.** Different *B. thetaiotaomicron* strain growth utilising BEV bound cobalamin as the only source (B12-BEV) and a cobalamin-free BEV control (BEV). Boxplots show four biological replicates of one BEV preparation. **C.** BEV release over time by different *B. thetaiotaomicron* mutant strains highlighting the role of BtuL. Dots show representative average 20*OD_600_ value change from inoculation; bars show BEV concentration in the supernatant as one standard deviation in 4 biological replicates. Note the difference in BEV release between 1 and 5 h of culture. **D.** *B. fragilis* utilisation of cobalamin bound to *B. thetaiotaomicron* BEVs.

Growth experiments for these strains were conducted across a range of cobalamin concentrations, including conditions where BEV-associated cobalamin was the sole source (Figure 7A). Notably, differences in growth were observed at low cobalamin concentrations that were not apparent at higher levels. Interestingly, the ∆13 .J2.H2.L mutant strain outperformed the wildtype strain under low cobalamin concentrations. A similar trend was observed when BEV-associated cobalamin served as the only source of cobalamin (Figure 7B).

Based on the enhanced performance of the ∆13 .J2.H2.L strain, the significant up-regulation of BtuL in BEVs and the absence of detectable cobalamin binding by BtuL, we hypothesised that BtuL might play a role in promoting BEV release. To test this, we measured BEV release over time in wildtype, ∆1234, and ∆*btuL B. thetaiotaomicron* strains (Figure 7C). Notably, both mutant strains produced fewer BEVs during the early growth phase compared with the wildtype strain. Interestingly, this difference was no longer observed in the later stages of growth.

In our previous work, we demonstrated that BEV-associated cobalamin is not bioavailable to an auxotrophic *Salmonella* strain [3]. To investigate whether BEV-bound cobalamin is accessible to closely related *Bacteroides* species, we tested its bioavailability in *Bacteroides fragilis* NCTC 9343, a strain that lacks most of the cobalamin uptake genes present in *B. thetaiotaomicron* (Supplementary Figure S4). Interestingly, this *B. fragilis* strain was able to utilise BEV-bound cobalamin (Figure 7D). However, BEVs produced by *B. fragilis* did not exhibit cobalamin-binding activity. This finding further supports the role of BtuJ as the key cobalamin-binding protein on BEVs, as BtuJ is absent in this particular *B. fragilis* strain.

## Discussion

**Figure 8:**
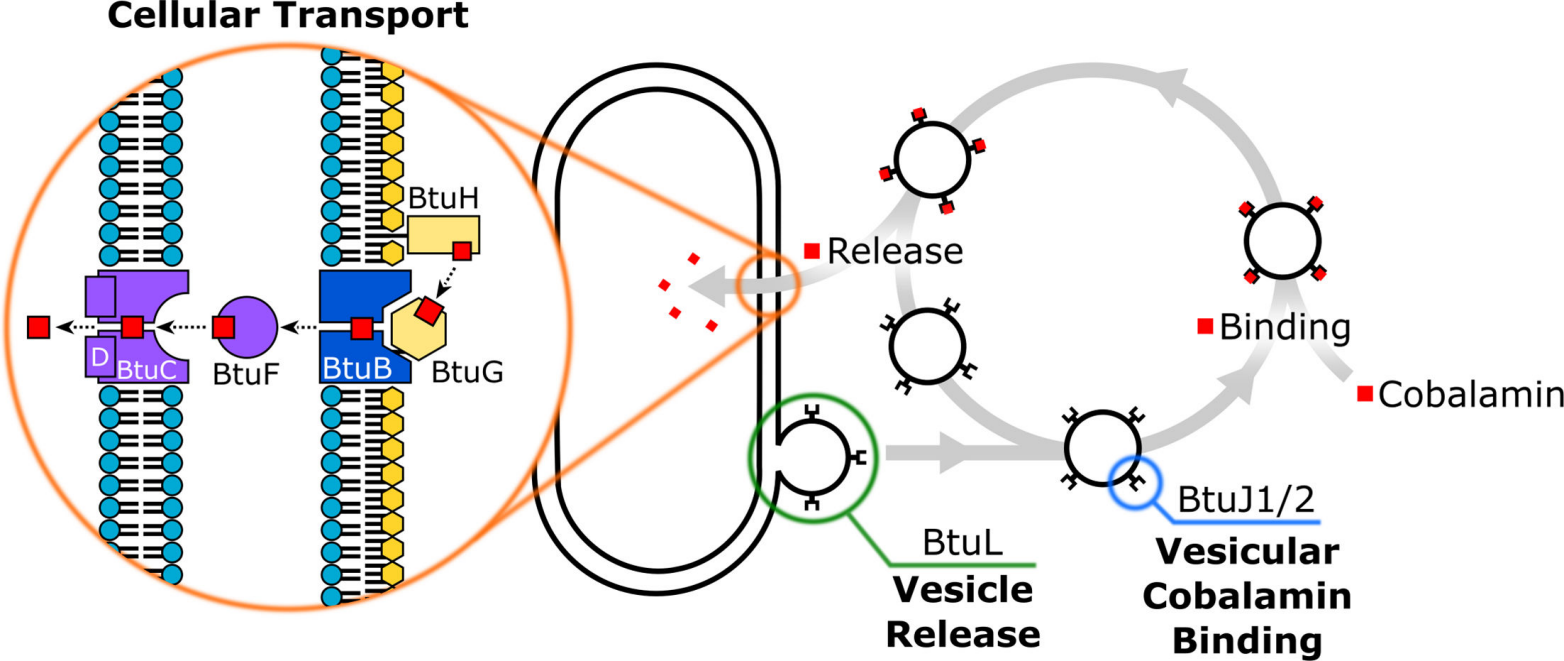
Revised model of cobalamin acquisition in B*. thetaiotaomicron*. The proposed mechanism based on this publication suggests that BtuL mediates extracellular vesicle release, while BtuJ1 and BtuJ2 serve as the primary factors responsible for cobalamin binding on the vesicles.

Bioinformatic analysis of the four cobalamin uptake operons in *B. thetaiotaomicron* VPI-5482 revealed the presence of an internal promoter in operon 1 and the absence of a riboswitch in operon 4. Recombinant promoter-reporter assays demonstrated that this internal promoter is not only active but also approximately an order of magnitude stronger than the primary promoter at the start of the operon. This suggests relatively low expression of *btuK1, btuK2* and *btuH1*. Despite the lack of an identifiable riboswitch in operon 4, its gene expression was regulated similarly to the other cobalamin uptake operons. This suggests significant sequence divergence from the defined cobalamin riboswitch or the presence of an alternative regulatory element. An ongoing question is how these operons are co-ordinated to allow for optimal corrinoid uptake, and how the range of encoded proteins promote the transfer of the nutrient from the environment to the cell. Figure 8 outlines a model in which multiple Btu proteins co-operate: ‘capture’ proteins intercept scarce cobalamin molecules in the extracellular milieu, channelling them towards the downstream transporter system.

The kinetic phenotypes we observe across the Btu protein family, ranging from association rates approaching diffusion limits to dissociation rates so slow they strain conventional measurement techniques, paint a picture of ultra-tight molecular recognition. Among these, BtuJ1, BtuJ2 and BtuK1 stand out as candidates for picomolar or even sub-picomolar binding to cyanocobalamin. If confirmed, such affinities place these proteins in the rarefied category of nature’s most exquisitely evolved ligand binders.

Crucially, our findings point to a central role for the BtuJ proteins in this network (Figure 8). Their extremely high affinities make them ideal candidates for BEV-mediated nutrient sequestration: by displaying BtuJ on the surface of BEVs, cells may effectively increase their environmental reach, capturing and concentrating scarce corrinoids at a distance from the cell surface. This interpretation aligns with recent evidence that BEVs function as competitive nutrient traps in microbial communities. Complementing this, BtuL has been implicated in promoting vesicle formation itself, suggesting a co-ordinated strategy in which BtuL drives vesiculation while BtuJ endows vesicles with exceptional nutrient-scavenging capacity (Figure 8). Together, these proteins may represent a previously underappreciated synergy between vesicle biology and cofactor acquisition.

The model highlights how BEVs can function in a similar fashion to siderophores, being released from cells to scavenge cobalamin in the environment by tight binding. Once bound, the vitamin is only made available to cells equipped with the appropriate receptor (Figure 8), with its release probably facilitated by other proteins. From a structural perspective, BtuJ and BtuG bind cobamide with the lower ligand loop exposed, facing outward from the binding pocket, whereas BtuH binds on the opposite side, with the lower ligand loop-oriented inwards [6,9]. This arrangement supports a mechanism in which cobalamin captured by BtuJ on BEVs is transferred to BtuH on the cell surface, and subsequently onto the BtuBG complex. Further evidence for this mechanism comes from the presence of a BtuGH fusion protein in operon 1. This model would allow for a reduced number of BtuBG complexes on the cell surface if BtuH is present to mediate cobamide capture. The similarity between the augmented β-jellyroll barrel and immunoglobulin β-barrel proteins, both using six loops to tightly bind ligands, has not gone unnoticed. While the immunoglobulin loops have evolved to accommodate a wide range of binding partners, the loops of the augmented β-jellyroll may have a far narrower range of binding partners, possibly restricted to cobamides.

From a biotechnology and synthetic biology perspective, these findings carry significant implications. For instance, the proteins can be used in affinity purification and capture, where ultra-tight Btu proteins could be deployed as affinity matrices for isolating cobalamin or cobamide analogues, reducing the cost of vitamin B_12_ production. Similarly, the proteins can be used in biosensor platforms, allowing the detection with picomolar sensitivity. Through metabolic engineering, Btu modules can be engineered in strains to enhance uptake and flux through B_12_-dependent pathways. Finally, BEV-based strategies leveraging BtuJ could inspire therapeutic applications, either to deliver cobalamin analogues selectively or to restrict cobalamin availability in competing microbes.

In summary, our results point to a two-layered innovation: the evolution of Btu proteins with some of the highest binding affinities known in biology, and their integration into BEV-based nutrient acquisition systems. Together, these strategies could provide a blueprint for engineering new cobalamin-based biotechnologies, from separation platforms and biosensors to vesicle-based delivery tools, while also shedding light on the ecological arms race for scarce cofactors in microbial communities.

## Methods

### Bacterial culture conditions

*E. coli* was grown in lysogeny broth (LB) containing 10g/l Tryptone; 5g/l Yeast Extract; NaCl 10 g/l at 37 °C at 200 rpm when grown in liquid culture. *B. thetaiotaomicron* was grown in an anaerobic cabinet (10% H2, 5% CO2, 85% N2) at 37°C with no agitation. Brain Heart Infusion supplemented with 4-μM hemin (BHIH) or Bacteroides Defined Media r6 (BDMr6) consisting of 100 mM potassium phosphate pH 7.8 (KH_2_PO_4_ 9.2 mM; K_2_HPO_4_ 90.8 mM), 15 mM NaCl, 8.5 mM (NH_4_)_2_SO_4_; 30 mM glucose; 100 µM MgCl_2_; 50 µM CaCl_2_; 2 mM L-Cysteine hydrochloride; 10 μM FeSO_4_; 400 µM L-methionine (or specified concentration of L-methionine/cobamide); 50 nM Protoporphyrin IX (prepared in 50% ethanol; 50 mM NaOH) was used to culture cells. All components purchased from Merck unless otherwise specified.

### Molecular biology

E. coli DH5α (NEB) used to construct expression plasmids; *E. coli* PIR1^+^ (ThermoFischer Scientific) used for integration and knockout vectors. Chemically competent *E. coli* cells were used with transformations carried out using heat-shock method; *B. thetaiotaomicron* transformations were carried out via conjugation using relevant *E. coli* donor strains as previously described (27). All primers and plasmids used are outlined in Supplementary Table S2 and Table S3; all plasmid sequence maps are also available as Supplementary Dataset 1. Plasmid sequences were confirmed using Sanger sequencing (Genewiz).

To generate the cobalamin promoter *B. thetaiotaomicron* reporter construct, a synthetic DNA fragment (Integrated DNA Technologies) containing a promoter (P.Bth_BT1830s); ribosome binding site (RBS.Bth_RBS7) and a codon optimised Nanoluciferase; flanked by terminators (T.BBa_B1001; T.BBa_B1007) was inserted into a single copy integration vector pNBU2_erm-TetR-P1T_DP-GH023 *AflII*/*BamHI* site yielding pIBATH.56 [27,28]. *BglII*/*NdeI* sites were encoded in the DNA fragment to allow for the replacement of promoter and RBS site. This site was used to insert DNA fragments corresponding to the promoter and RBS sites generated by PCR (Phusion; NEB) using outlined primers (Supplementary Table S1 and Table S2; Supplementary Dataset S1).

To generate plasmids for protein expression in *E. coli,* the soluble parts of the genes of interest, lacking the predicted lipoprotein motif as identified using SignalP 6.0 [11], were amplified via PCR using primers containing restriction site overhangs outlined in Supplementary Figure S2). The amplified fragments were inserted into a modified pET14b vector (novel *SpeI* site between the *NdeI* and terminator) *NdeI/SpeI* site resulting in a construct encoding for an N-terminus hexahistidine tagged protein produced via the T7 promoter (Supplementary Figure S2; Supplementary Dataset S 1).

To generate single gene expression plasmids for *B. thetaiotaomicron*, pGH117 was used as the vector [29]. The *Pci*/*BamHI* cloning site was used to introduce a synthetic promoter (P.Bth_BT1830s) and ribosome binding site (RBS.Bth_RBS7) followed by an *NdeI*/*SpeI* cloning site containing an ORF (not relevant to this study) with artificial bidirectional terminators (BBa_B1001; BBa_B1007) flanking the operon yielding pBATH.03 [28]. PCR was used to amplify genes of interest using *B. thetaiotaomicron* VPI-5482 DNA and primers (Integrated DNA Technologies) designed with *NdeI/SpeI* flanking restriction sites, which were used to insert the generated fragments into the pBATH.03 vector *NdeI/SpeI* site (Supplementary Figure S2 and S3; Supplementary Dataset S 1). The generated plasmids were used to transform *B. thetaiotaomicron* strain lacking all three cobalamin uptake operons (*Δlocus1 Δlocus2 Δlocus3 Δtdk* strain) [7].

*B. thetaiotaomicron* VPI-5482 mutant strains were generated using a previously reported method [30]. Plasmid for the cross-over (Supplementary Figure S3; Supplementary Dataset S 1) was generated via insertion of a fragment generated by overlap extension PCR. Primers corresponding to downstream (Down), or upstream (Up) fragments were used to amplify DNA upstream and downstream of the target DNA (Supplementary Table S2; Supplementary Dataset S 1). These fragments were combined using overlap extension PCR. Primers contained engineered restriction enzyme sites for *BamHI* (Up) and *PstI* (Down), which were used to insert the fragment into pLGB13 [30]. Post conjugation, individual recovered colonies were grown overnight in BHIH containing gentamicin (200 µg/ml) and erythromycin (25 µg/ml) and streaked out on BHIH agar plates containing gentamicin (200 µg/ml) and anhydrotetracycline (100 ng/ml). Individual colonies were then tested using PCR for the loss of the target DNA segment. To generate multiple edit strains, this process was repeated.

### Promoter characterisation

*B. thetaiotaomicron* VPI-5482 cobalamin promoter reporter strains were generated via transformation with the generated reporter plasmids (pIBATH.104–108; Supplementary Table S2; Supplementary Dataset S 1). Two colonies for each transformation were selected and cultured in duplicate overnight in 5 ml of BDMr6. Then, 10 µl was used to inoculate 200 µl of BDMr6 containing either 200 µM L-methionine; 500 pM cyanocobalamin (vitamin B_12_); 500 pM pseudocobalamin (Ps); or 500 pM methylbenzimidazole cobamide (5MB) in a flat-bottom 96 well plate (bio-one cellstar; Greiner), sealed with adhesive gas permeable seals to limit evaporation, overnight. Nanoluciferase signal was quantified using Nano-Glo® Luciferase Assay System (Promega) and adjusted to cell density as estimated by optical density at 600 nm (OD_600_). Readings were carried out using the CLARIOstar Plate Reader (BMG Labtech) with standard instrument settings.

### Protein expression

*E. coli* BL21(DE3) cells were used for protein production. Transformants selected using 100 µg/ml of ampicillin. Single colonies were used to inoculate 50 ml of LB (100 µg/ml ampicillin) and cultured overnight at 37°C. Then, 8 ml of the overnight culture was transferred to 800 ml of fresh media and cultivated until the culture reached the mid-log phase, measured with an OD_600_ value of 0.6. At this point, the culture was cooled down and induced with IPTG (1 mM). The induced culture was incubated at 18°C overnight at 200 rpm. Bacteria were collected by centrifugation, and the obtained pellets were kept frozen at -20 °C until required.

### Protein purification

Bacterial pellet resuspended in 100 ml of binding buffer (20 mM Tris-HCl, pH 7.5, 500 mM NaCl, 30 mM imidazole, 1 mM TCEP), supplemented with complete protease inhibitor cocktail tablets (Roche), and sonicated on ice. The obtained lysates were clarified by centrifugation (16,000×g, 30 min, 4°C), followed by ultracentrifugation (80,000×g, 30 min, 4°C), and supplemented with 1 ml of cyanocobalamin stock solution (10 mg/ml). The resulting supernatant was passed five times through a pre-packed HisTrap HP column containing 5 ml of chelating Sepharose (Cytiva). Subsequently, the column was washed with 100 ml of binding buffer, and the target protein was eluted in 5 ml fractions with elution buffer (20 mM Tris-HCl, pH 7.5, 500 mM NaCl, 500 mM imidazole, 1 mM TCEP). Following protein elution, the elution fractions containing the highest amount of total protein (estimated by absorbance at 280 nm) were combined, concentrated by centrifugation to a final volume of 5 ml (Vivaspin 20; 10 kDa cut-off; Sartorius), and applied to a size-exclusion chromatography column (Superdex 200, 16/60) at a flow rate of 1.5 ml/min in SEC buffer (20 mM Tris-HCl, pH 7.5, 150 mM NaCl, 1 mM TCEP). The fractions corresponding to the expected elution volumes for the purified proteins were collected, flash-frozen in liquid nitrogen and stored at −80°C.

### Stopped-flow experiments and fluorescence spectroscopy

All measurements were conducted using a Hi-Tech Scientific SF-61 single mixing stopped-flow system at 20°C in SEC buffer, employing 290 nm LEDs for tryptophan fluorescence excitation. Tryptophan fluorescence was monitored through a WG 320 filter. Data collection and analysis were performed with software provided by Hi-Tech and followed standard collection and analysis procedures as set out below [20]. The presented transients represent an average of 10–14 consecutive shots from the stopped-flow apparatus, ensuring a noise-to-signal ratio as close as possible to 10%. Any transients exhibiting drift between shots were excluded. The quoted concentrations reflect those in the reaction chamber post-mixing, which entails a two-fold dilution from pre-mixing concentrations. Standard working volumes of 1 ml were used. Stopped-flow data were fitted to a single exponential model through a least-squares curve fit using Hi-Tech software. A large excess of cobalamin was used to maintain pseudo first order conditions facilitating the calculation of the observed binding rate constant (*K*obs) from the exponential change in fluorescence. Plots of *K*obs vs (cobalamin) yielded a straight-line plot (*K*obs = *K*on (cobalamin) + *K*off) from which Kon was estimated from the slope of the plot. In principle, *K*off (and hence *K*d = *K*off*/K*on) can be estimated from the intercept value, but *K*d was too tight, and the intercept value was not accurately defined. In some instances, the *K*off value could be estimated from a displacement experiment in which cobalamin was chased from its complex with the protein by addition of a large excess of a second cobalamin binding protein.

### Surface plasmon resonance

SPR experiments were performed using an LSA-XT instrument (Carterra). The sensor surface and sample deck were maintained at 20°C and 15°C, respectively. HC200M and HC30M sensor chips were utilised for Ado-Cbl and cyanocobalamin, respectively. Single-channel fluidics were primed with HBSTE buffer (10 mM HEPES, pH 7.4, 0.5 mg/ml BSA, 150 mM NaCl, 0.05% Tween 20, and 3 mM EDTA) as the running buffer. Prior to immobilisation, the chip was conditioned by sequential 1 min injections of 50 mM NaOH and 1 M NaCl.

Each protein was prepared at a concentration of 50 μg/ml in 10 mM sodium acetate, adjusted to one of four pH values (4.0, 4.5, 5.0, and 5.5). The chip surface was activated using a freshly prepared activation mixture (1:1:1 ratio of 100 mM 2-Morpholinoethanesulfonic acid (MES), pH 5.5, 100 mM sulfo-N-hydroxysuccinimide (Sulfo-NHS), and 400 mM 1-ethyl-3-(3-dimethylaminopropyl) carbodiimide hydrochloride (EDAC HCl)). Activation was carried out with a 7 min injection, followed by direct coupling of proteins onto the chip surface using the multi-channel fluidic module via a 10 min injection. The surface was then quenched with 0.5 M ethanolamine, pH 8.5, for 7 min. Following immobilisation, six HBSTE buffer injections were performed to stabilise the baseline.

For analyte binding studies, cobamides were injected as a 3-fold dilution series ranging from 1.2 to 100 nM. Injection cycles consisted of a 60 s baseline stabilisation, followed by a 7 min association phase and a 20 min dissociation phase.

SPR data for BtuF3 and BtuH1 were analysed using a global Y-alignment method due to their relatively fast dissociation rates, whereas BtuGH, BtuG2, BtuG3 and BtuK1 were analysed using non-regenerative kinetic modelling. Standard referencing was performed by subtracting signals from adjacent empty reference spots, followed by double referencing using a leading blank injection.

Non-regenerative kinetics were employed, ensuring that a full analyte titration series was performed without regeneration between injections. For ligands exhibiting slow dissociation (i.e., analyte binding did not return to baseline within each cycle), serial Y-alignment was applied using the baseline of the first analyte injection, followed by baseline cropping. The resulting serially Y-aligned data were fitted using a 1:1 Langmuir binding model with the T₀ float option enabled. Ligands with faster dissociation rates were analysed using global Y-alignment and fitted to the standard 1:1 Langmuir binding model.

### Crystallisation and data collection

BtuJ1 protein was concentrated to 40 mg/ml by ultrafiltration (Vivaspin 20 devices, 10 kDa cut-off, Sartorius). The initial screening for crystallisation conditions was performed using commercially available sparse matrix crystallisation screening kits (Molecular Dimensions) with the sitting drop vapour diffusion approach in standard 96-well crystallisation plates. The total drop size was 0.4 µl, consisting of equal volumes of protein stock solution and mother liquor, with a reservoir solution volume of 100 µl. The plates were incubated at 20°C. Further optimisation of screening conditions was used in the hanging drop vapour diffusion method in standard 24-well plates. The total drop size was 2.0 µl, consisting of equal volumes of protein stock solution and mother liquor, with a reservoir solution volume of 1.0 ml. The mother liquor composition was determined based on results from the sparse matrix screening experiments and was as follows: trisodium citrate: 0.05/0.1/0.2/0.3 M, pH 3.5; PEG 6000: 15/18/21/24/29/33% (w/v). The plates were stored at 20°C. For vitrification, the cryoprotectant consisting of the corresponding mother liquor solutions containing 20% glycerol (v/v) was used. Prior to X-ray data collection, the mounted crystals were stored in standard Uni-pucks (Molecular Dimensions) in liquid nitrogen. BtuJ2, protein crystals were taken directly from the screening plate of structure 2 screen 29 (2M ammonium sulfate, 0.1M sodium citrate pH 5.6, 0.2M potassium sodium tartrate tetrahydrate) and cryo-protected with mother liquor augmented with 20% glycerol. Diffraction data were collected at ESRF beamline ID23-2 (ESRF, Grenoble).

### X-ray structure determination

The X-ray diffraction data were processed using auto-processing pipelines at ESRF. The space group was determined with POINTLESS [31] and data merged using AIMLESS [32]. The preliminary model was built using the AlphaFold MR workflow in the CCP4i suite [33] and improved using ModelCraft [34], followed by rounds of manual building in Coot [35] and refinement with REFMAC [36]. MolProbity [37] was used to validate the protein geometry, and PyMOL [38,39] was used for the visualisation of the protein structure. The co-ordinates and structure factors of BtuJ1 and BtuJ2 in complex with cyanocobalamin were deposited in the Protein Data Bank with identifiers 9FCT and 9I2L, respectively.

### BEV preparation and proteomic analysis

*B. thetaiotaomicron* VPI-5482 cells were grown overnight from frozen stocks in 5 ml BDMr6 in triplicate. A 200 µl of these cultures was used to inoculate 5 ml BDMr6 with either 400 µM L-methionine or 1 µM cyanocobalamin and grown for 5 h. A 4 ml of these cultures was then transferred into 500 ml BDMr6 with either 400 µM L-methionine or 1 µM cyanocobalamin and grown for 14 h. BEV purification and proteomic analysis were carried out as described below.

To lyse the cells/BEVs, sodium dodecyl sulphate (SDS) was added to 2% followed by boiling and vortexing. Proteins were precipitated with methanol/chloroform and the protein pellets were resuspended in 200 µl of 2.5% sodium deoxycholate (SDC) in 0.2 M EPPS-buffer pH 8, and vortexed under heating. Protein concentration was estimated using a BCA assay and 100 µg of protein per sample was treated with dithiothreitol and iodoacetamide to alkylate cysteine residues and digested with trypsin in the SDC buffer according to standard procedures. After the digest, the SDC was precipitated by adjusting to 0.2% trifluoroacetic acid (TFA), and the clear supernatant was subjected to C18 SPE (Reprosil, Dr. Maisch GmbH). Peptide concentration was further estimated by running an aliquot of the digests on LCMS. Isobaric labelling was performed using a TMT™ 6plex kit (Lot VF291489, ThermoFisher Scientific) according to the manufacturer’s instructions with slight modifications; approx. 100 µg of the dried peptides were dissolved in 90 µl of 0.2 M EPPS buffer/10% acetonitrile, and 250 µg TMT reagent dissolved in 22 µl of acetonitrile was added. Samples were assigned to the TMT channels in the following order: Experiment qib43rj (BEV samples): channels 126, 127 and 128 assigned to condition M, 129, 130 and 131 to condition B; Experiment qib48rj (cell samples): channels 126, 128 and 130 assigned to condition M; channels 127, 129 and 131 to condition B.

After 2 h incubation, aliquots of 1 µl from each sample were combined in 400 µl of 0.2% TFA, desalted, and analysed on the mass spectrometer (same method as for TMT, see below, but without RTS) to check labelling efficiency and estimate total sample abundances. The main sample aliquots were quenched by adding 8 µl of 5% hydroxylamine and then combined to roughly level abundances and desalted using a C18 Sep-Pak cartridge (200 mg, Waters). The eluted peptides were dissolved in 500 µl of 25 mM NH_4_HCO_3_ and fractionated by high pH reversed phase HPLC. Using an ACQUITY Arc Bio System (Waters), the samples were loaded to an XBridge® 5 µm BEH C18 130 Å column (250 × 4.6 mm, Waters). Fractionation was performed with the following gradient of solvents A (water), B (acetonitrile) and C (25 mM NH_4_HCO_3_ in water) at a flow rate of 1 ml min^-1^: solvent C was kept at 10% throughout the gradient; solvent B: 0–5 min: 5%, 5–10 min: 5–10%, 10–80 min: 10–45%, 80–90 min: 45–80%, followed by 5 min at 80% B and re-equilibration to 5% for 24 min. Fractions of 1 ml were collected and concatenated by combining fractions of similar peptide concentration to produce 16 final fractions for experiment qib43rj (BEV samples) and 21 final fractions for experiment qib48rj (cell samples) for MS analysis. Aliquots were analysed by nanoLC-MS/MS on an Orbitrap Eclipse™ Tribrid™ mass spectrometer coupled to an UltiMate^®^ 3000 RSLCnano LC system (Thermo Fisher Scientific). The samples were loaded onto a trap cartridge (Pepmap Neo, C18, 5 um, 0.3 × 5 mm, Thermo) with 0.1% TFA at 15 µl min^-1^ for 3 min. The trap column was then switched in-line with the analytical column (nanoEase M/Z column, HSS C18 T3, 1.8 µm, 100 Å, 250 mm × 0.75 µm, Waters) for separation using the following gradient of solvents A (water, 0.1% formic acid) and B (80% acetonitrile, 0.1% formic acid) at a flow rate of 0.2 µl min^-1^ : 0–3 min 3% B (parallel to trapping); 3–10 min linear increase B to 8%; 10–105 min increase B to 50%; 105–113 min linear increase B to 99%; keeping at 99% B for 3 min and re-equilibration to 3% B. Data were acquired with the following parameters in positive ion mode: MS1/OT: resolution 120K, profile mode, mass range *m/z* 400–1800, AGC target 4e^5^, max inject time 50 ms; MS2/IT: data-dependent analysis with the following parameters: 3 s cycle time Rapid mode, centroid mode, quadrupole isolation window 0.7 Da, charge states 2–5, threshold 1.9e^4^, CID CE = 35, AGC target 1e4, max. inject time 70 ms, dynamic exclusion 1 count for 15 s mass tolerance of 7 ppm; MS3 synchronous precursor selection (SPS): 10 SPS precursors, isolation window 0.7 Da, HCD fragmentation with CE = 65, Orbitrap Turbo TMT and TMTpro resolution 30 k, AGC target 1e5, max inject time 105 ms, Real Time Search (RTS): protein database *Bacteroides thetaiotaomicron* (uniprot.org, March 2022, 7482 entries), enzyme trypsin, 1 missed cleavage, oxidation (M) as variable, carbamidomethyl (C) and TMT as fixed modifications, precursor tolerance 6 ppm, Xcorr = 1, dCn = 0.05.

The acquired raw data were processed and quantified in Proteome Discoverer 3.0 (Thermo) using the incorporated search engines Comet and CHIMERYS (MSAID, Munich, Germany). The processing workflow for both engines included recalibration of MS1 spectra (RC), reporter ion quantification by most confident centroid (20 ppm) and a search on the *Bacteroides thetaiotaomicron* protein database and a common contaminants database. For CHIMERYS, the Top N Peak Filter was used with 20 peaks per 100 Da and the inferys_2.1_fragmentation prediction model was used with fragment tolerance 0.5 Da, enzyme trypsin with 1 missed cleavage, variable modification oxidation (M), fixed modifications carbamidomethyl (C) and TMT6plex on N-terminus and K. For Comet, the version 2019.01 rev0 parameter file was used with default settings except precursor tolerance set to 6 ppm and trypsin missed cleavages set to 1. Modifications were the same as for CHIMERYS.

The consensus workflow included the following parameters: assigning the four replicates/channels as described above per condition, only unique peptides (protein groups) for quantification, intensity-based abundance, TMT channel correction values applied (VF291489), co-isolation/SPS matches thresholds 50%/70%, normalised CHIMERYS Coefficient Threshold 0.8, normalisation on total peptide abundances, protein abundance-based ratio calculation, missing values imputation by low abundance resampling, hypothesis testing by *t*-test (background based), adjusted *P* value calculation by BH method.

### *Bacteroides* growth assay

For single gene rescue experiments, *B. thetaiotaomicron* VPI-5482 *Δtdk Δlocus1 Δlocus2 Δlocus3* [7] strain was transformed with single gene expression plasmids (pBATH.68–83; Supplementary Table S 3). Single colonies were selected and grown in 15 ml of BHIH containing 400 µM L-methionine, 250 nM cyanocobalamin and 25 µg/ml erythromycin overnight. The cells were separated using centrifugation at 3,000×g for 20 min and the supernatant was filtered via a 0.22 µm PES filter. The BEVs were then separated and concentrated using a VivaSpin6 100 kDa PES centrifugal filter followed by 5 washes with 5.5 ml of PBS, concentrating to the minimal volume between washes. The final concentrated solution was resuspended in 15 ml of BDMr6, containing no L-methionine, and filter sterilised using a 0.22 µm PES filter. Then, 1 ml of the resulting BEV containing media in 24-well plate (bio-one cellstar; Greiner) was inoculated with 50 µl of *B. thetaiotaomicron* VPI-5482 *Δtdk* pre-cultured in BDMr6 sealed with adhesive gas permeable seals to limit evaporation. A negative control was included for each BEV containing BDMr6 condition and a positive control containing 200 µM L-methionine was used. Plates were incubated overnight, and cell growth was evaluated by OD_600_. Note that *B. thetaiotaomicron* VPI-5482 must be pre-cultured in BDMr6 with L-methionine and lacking any source of cobalamin for several generations for the assay to work. This ensures that any stored cobalamin is depleted prior to the bioassay.

For *ΔbtuJ1* and *ΔbtuJ2* experiments, wildtype *B. thetaiotaomicron* VPI-5482 was used as the strain to generate knockouts and as the bioassay test strain. *B. thetaiotaomicron* VPI-5482 Δ1234 strain, specifically lacking all of the uptake operons, was also constructed as *B. thetaiotaomicron* VPI-5482 *Δtdk Δlocus1 Δlocus2 Δlocus3* strain, which contains additional gene knockouts found between operons 3 and 4. Two mutant colonies were selected for each strain and cultured in 15 ml of BDMr6 containing 400 µM L-methionine to mid-log phase. Further assay steps were carried out as above, except cyanocobalamin was added to 250 nM to the filtered supernatant prior to BEV preparation. Assays were carried out in 1 ml in 48-well plates (bio-one cellstar; Greiner) sealed with adhesive gas permeable seals to limit evaporation. Plates were incubated overnight and OD_600_ readings were taken indicating cell growth. Each BEV preparation was tested in four replicates.

For cobalamin uptake operon mutant analysis, two separate colonies of the different mutant strains were inoculated in duplicate (four replicates) in 5 ml of BDMr6 and grown overnight. 50 µl of was then used to inoculate 1 ml of BDMr6 in 48 well plates (as above), where L-methionine was replaced with different amounts of cyanocobalamin or BEVs prepared from wildtype *B. thetaiotaomicron* VPI-5482 as in the *ΔbtuJ1* and *ΔbtuJ2* experiment. Cell growth was evaluated as above.

BEV release overtime experiments were carried out based on the previously described method with slight modifications [10]. Two colonies for each strain were selected and used to inoculate 5 ml of BDMr6 (200 µM L-methionine) and grown overnight. Then, 1 ml was then sub-cultured into 300 ml of BDMr6 (200 µM L-methionine) and grown for 16 h. Cultured were then centrifuged in sterile bottles pre-equilibrated in anaerobic cabinet at 6,600 g at 21°C for 10 min. Pelleted cells resuspended in BDMr6 and used to inoculate 300 ml of BDMr6 to starting OD_600_ of 0.25. Lipid concentration evaluated using FM4-64 dye-based assay [16].

*B. fragilis* NCTC 9343 BEV utilisation was evaluated using the bioassay as described for *ΔbtuJ1* and *ΔbtuJ2* experiments.

## Supplementary material

online supplementary material 1

## Data Availability

Supplementary datasets are available in the figshare repository upon publication. Supplementary Dataset 1: doi.org/10.6084/m9.figshare.29716061 Supplementary Dataset 2: doi.org/10.6084/m9.figshare.29716202 Supplementary Dataset 3: doi.org/10.6084/m9.figshare.29716250 The authors confirm that all data supporting the findings of this study are available within the article, repository and is also available from the corresponding authors on reasonable request.

## Open Access

This article has been published open access under our Subscribe to Open programme, made possible through the support of our subscribing institutions, learn more here: https://portlandpress.com/pages/open_access_options_and_prices#conditional

## Abbreviations

BEVs: bacterial extracellular vesicles
LB: lysogeny broth
SPR: surface plasmon resonance
